# Editorial: Discovery, biosynthesis, regulation, transport, release and engineering of plant natural products

**DOI:** 10.3389/fpls.2022.1017808

**Published:** 2022-09-02

**Authors:** Wei Zhou, Milen I. Georgiev, Pan Liao

**Affiliations:** ^1^Laboratory for Core Technology of TCM Quality Improvement and Transformation, School of Pharmaceutical Sciences, Zhejiang Chinese Medical University, Hangzhou, China; ^2^Laboratory of Metabolomics, Institute of Microbiology, Bulgarian Academy of Sciences, Plovdiv, Bulgaria; ^3^Center of Plant Systems Biology and Biotechnology, Plovdiv, Bulgaria; ^4^Department of Biology, Hong Kong Baptist University, Kowloon Tong, Hong Kong SAR, China; ^5^Institute of Bioresource and Agriculture, Hong Kong Baptist University, Kowloon Tong, Hong Kong SAR, China; ^6^State Key Laboratory of Agrobiotechnology, The Chinese University of Hong Kong, Shatin, Hong Kong SAR, China; ^7^Department of Biochemistry, Purdue University, West Lafayette, IN, United States; ^8^Purdue Center for Plant Biology, Purdue University, West Lafayette, IN, United States

**Keywords:** natural products, biosynthesis, regulation, transcriptomics, metabolomics, transferases

## Introduction

Humans have utilized natural products derived from plants as medicines for 1,000's of years. A number of plant natural products have economic and pharmaceutical value and are essential for the scent of flowers, the flavor of fruits, and for the protection of plants against stress. Although progress has been made in the discovery of enzymes and genes in plants, the biosynthetic pathways for many natural products remain unclear. In this Research Topic of collection, we are interested in studies on the discovery, biosynthesis, regulation, transport, release, and engineering of plant natural products.

## Discovery, biosynthesis, and modification of plant natural products

Correlation analysis of gene expression and metabolite accumulation has been shown to be useful for the discovery of novel genes for the biosynthesis of terpenoids. Wang et al. identified a sesquiterpene synthase (MrTPS3) for the biosynthesis of β-caryophyllene from Red Bayberry (*Morella rubra*) and a possible monoterpene synthase (MrTPS20) for α-pinene formation through combinational transcriptome and volatile organic compound analyses of samples from four cultivars during fruit development coupled with weighted gene co-expression network analysis. As a reaction two steps downstream of the 15-carbon intermediate farnesyl pyrophosphate in the biosynthesis of sesquiterpenes and triterpenes, Ye et al. identified an oxidosqualene cyclase from *Gynostemma longipes*. This enzyme was demonstrated to catalyze the conversion of 2,3-oxidosqualene to dammarenediol-II in yeast, by integrating metabolomic and transcriptomic analyses as well as Pearson correlation analysis between triterpene content and gene expression.

Acylation is a general and important modification of plant secondary metabolites. Plant BAHD acyltransferases include a big family consisting of members involved in the catalysis of transferring coenzyme A-activated donors to diverse acceptor molecules. Choi et al. identified a triterpene acetyltransferase (LsTAT1) from Lettuce (*Lactuca sativa*), which belongs to the membrane-bound *O*-acyltransferase (MBOAT) family, and found that it can specifically convert free pentacyclic triterpenes to their acetates when characterizing its function in yeast and tobacco. However, it was not able to convert free sterols to their acetates, indicating that LsTAT1 is functionally different from sterol acyltransferases. While Qiang et al. identified a coniferyl alcohol acyltransferase (CFAT) out of 37 BADH genes, from the transcriptome datasets of *Schisandra chinensis*. *In vitro* enzyme assays of the recombinant ScBAHD1 revealed that it displayed acyltransferase activity on broad alcohol substrates including coniferyl alcohol using acetyl-CoA as the acetyl donor, but ScBAHD1 cannot use compounds with phenol hydroxyl as substrates.

Genes involved in other modifications such as detoxification and C-3 glycosylation have also been reported. Zhang et al. found two *Arabidopsis thaliana* glutathione-*S*-transferases (GSTs), GSTF11, and GSTU20, that play important role in the biosynthesis of aliphatic glucosinolates, which are sulfur-rich secondary metabolites mainly exist in *Brassicale* plants, known as defense compounds and display anticancer, antioxidant and antimetastatic activities that are beneficial to human health. Anthocyanins are major contributor to flower and fruit color, Sun et al. found that anthocyanidin 3-*O*-glycosides were the major anthocyanins in flowers of *Rhododendron delavayi*, suggesting that flavonoid 3-*O*-glycosyltransferase (3GT) should play an important role for the color formation of *R. delavayi*. Hence, the authors performed correlation analysis of gene expression and anthocyanin accumulation during floral development and identified two potential 3GT candidates, Rd3GT1, and Rd3GT6. Further *in vitro* and *in vivo* experiments demonstrated that they both catalyze the addition of UDP-sugar to the 3-OH of anthocyanidin, with cyanidin as the most efficient substrate and UDP-Gal as their preferred sugar donor.

Hololectins appeared important in pathogenic and stress responses in plants, while also raising health concerns in humans. To understand better on the biosynthesis of cleavable and non-cleavable hololectins in cereals, Loo et al. identified nine hevein-like peptides from Oat (*Avena sativa*), avenatide aV1-aV9. Bioinformatic analysis of these peptides showed that asparaginyl endopeptidase (AEP)-susceptible linker length determines the maturation of peptides. Detailed analysis of avenatide aV1 showed that it had eight cysteine residues and could generate a structurally compact, metabolic-resistant cystine-knotted framework containing a well-defined chitin-binding site. Moreover, antimicrobial assays revealed that avenatide aV1 display anti-fungal property.

## Regulation of plant natural product biosynthetic pathways at the transcriptional level and alternative splicing at the post-transcriptional level

To achieve better outcomes for future metabolic engineering of valuable natural products, it is necessary to better understand how the biosynthesis of certain natural products is regulated. The regulation of a biosynthetic pathway could be mediated by a transcriptional factor at the transcriptional level and by other mechanisms at the post-transcriptional level such as alternative splicing (AS). For instance, an MYB transcription factor MYB113 from eggplant (*Solanum melongena* L.) has been found to be a key regulator for the anthocyanin composition variation and color diversity in the peels of different cultivars of eggplant (Yang et al.). Besides transcription factors, flavonoid biosynthesis can also be regulated by plant hormone signals such as ethylene, methyl jasmonate and auxin. He et al. recently reported that the overexpression of two *CtACO3s*, which are key enzyme genes for the ethylene signaling pathway in safflower (*Carthamus tinctorius* L.), enhanced the production of flavonoids such as flavonols and quinochalcones, respectively. Regarding AS, transcriptomic data of 36 samples of *Salvia miltiorrhiza* at twelve developmental stages were analyzed to investigate its genome wide AS events (Li et al.). The results showed that some physiological and molecular events including gravity response, lateral root formation, mitogen-activated protein kinase cascade and RNA splicing regulation were highly influenced by AS at the early seedling stage. Moreover, AS events were found to frequently exist for genes in the tanshinone and phenolic acid biosynthetic pathways at the early seedling stage, and a co-expression network including 521 highly expressed AS genes were identified, providing a better understanding of the variable programming of AS. He et al. experimentally demonstrated that *CtACO3-2*, which is a splice variant of *AtACO3*, lacked 5' coding sequences and encoded a protein that localized to both the cytoplasm and nucleus, while *CtACO3-1* encoded protein was targeted to cytoplasm only. Further analysis of CtACO3-2 using yeast two-hybrid, BiFC and glutathione *S*-transferase pull-down assays showed that CtACO3-2 interacted with *C. tinctorius* CSN Subunit 5 (CtCSN5a). CtCSN5a can interact with COI1, which is a subunit of SCF E3 ubiquitin ligase encoding an F-box protein and is required for jasmonic acid responses. These results suggest that *CtACO3-2* overexpression increases flavonoid accumulation by activating the expression of genes in flavonoid biosynthesis through CtbHLH3 and related JA signaling.

Moreover, two excellent review articles were covered on the Research Topic of the collection. First, the biosynthetic pathway for xanthones, which are secondary metabolites with pharmacological properties including antidiabetic, antitumor, and antimicrobes, has been comprehensively updated till 2021 (Remali et al.). It is worth noting that the biosynthetic pathways for downstream xanthone derivatives such as gambogic acid, swertianolin, gentisin, α-mangostin, mangiferin, and hyperixanthone A have also been proposed, which would be useful for future pathway mining of these compounds. In the second review, given that the major component of Mint essential oils is monoterpenes such as menthol are useful in the medical, pharmaceutical, cosmetic, and cleaning product industries due to their excellent biological properties. Fuchs et al. summarized the latest progress in metabolic engineering of monoterpene oils in Mint plants and future perspectives to further improve menthol and carvone was presented.

In summary, the collection of articles on this Research Topic provides updated information about the biosynthesis, modification, regulation, and metabolic engineering of plant natural products, which is useful for future improvement of value-added plant natural products through metabolic engineering and synthetic biology.

## Author contributions

All authors listed have made a substantial, direct, and intellectual contribution to the work and approved it for publication.

## Funding

Research of the topic editors was supported by the Department Start-up fund of Hong Kong Baptist University (BIOL-22-23-01; to PL), the European Union's Horizon 2020 research and innovation programme, project PlantaSYST (SGA No 739582 under FPA No. 664620), and the BG05M2OP001-1.003-001-C01 project, financed by the European Regional Development Fund through the Science and Education for Smart Growth Operational Programme (to MG) and the Key Scientific and Technological Grant of Zhejiang for Breeding New Agricultural Varieties (2021C02074-3; to WZ).

## Conflict of interest

The authors declare that the research was conducted in the absence of any commercial or financial relationships that could be construed as a potential conflict of interest.

## Publisher's note

All claims expressed in this article are solely those of the authors and do not necessarily represent those of their affiliated organizations, or those of the publisher, the editors and the reviewers. Any product that may be evaluated in this article, or claim that may be made by its manufacturer, is not guaranteed or endorsed by the publisher.

